# No utilization without subjective need – a mixed-methods study on guideline-compliant mental health care for individuals with depressive symptoms

**DOI:** 10.1186/s12913-026-15562-3

**Published:** 2026-09-10

**Authors:** Anna Katharina Reinhold, Elena Brushinski, Anna Levke Brütt

**Affiliations:** 1 Department of Health Services Research, Faculty of Medicine and Health Sciences, Carl von Ossietzky University Oldenburg, Oldenburg, Germany; 2 Department of Medical Psychology, University Medical Centre Hamburg-Eppendorf, Hamburg, Germany

**Keywords:** Perceived need, Depression, Incongruence of needs, Needs-based adequacy of care

## Abstract

**Background:**

The reasons why individuals with depressive disorders do not seek professional help are multifaceted and include illness-related, attitudinal, and structural barriers. While subjective need can facilitate help-seeking, its absence may act as a barrier. However, treatment guidelines primarily rely on objective need, defined by diagnosis, and do not address potential discrepancies between objective and subjective need. This study aimed to examine the frequency of congruent and incongruent mental health service use and to explore the conditions and reasons underlying incongruence between objective need, subjective need, and service use.

**Methods:**

This mixed-methods study used a sequential explanatory design based on a population-based sample of individuals with at least mild depressive symptoms (PHQ-9 ≥ 5). Quantitative data from telephone interviews conducted at baseline (T0) and 12 months later (T1) were used to assess objective need, subjective need, and mental health service use, and to determine whether service use was congruent or incongruent with guideline recommendations. Participants with different need-use patterns were then selected through theoretical sampling for subsequent qualitative interviews exploring the conditions and reasons underlying these patterns. Further cases were selected to refine, elaborate, or challenge categories developed through the Grounded Theory analysis according to Corbin and Strauss.

**Results:**

The analysis sample for descriptive analyses comprised 456 participants. Both the quantitative and qualitative data indicate that subjective need is a key predictor of help-seeking behavior. Seeking professional help in the absence of a perceived need is highly unlikely. Qualitative findings (*n* = 23) suggest that a dichotomous classification of subjective need (yes/no) is too simplistic and does not adequately reflect the reality of mental health care utilization. A typology of subjective need was therefore developed, identifying six types.

**Conclusions:**

This typology offers a framework for tailoring care more precisely to individuals’ perceived need. Our findings highlight the importance of considering subjective and objective need in parallel when evaluating service utilization, particularly in cases of incongruence. This requires a shift from a predominantly objective need-based approach toward a more comprehensive perspective that considers both subjective and objective need alongside barriers, prior treatment experiences, social support, and coping strategies.

**Supplementary Information:**

The online version contains supplementary material available at https://doi.org/10.1186/s12913-026-15562-3.

## Introduction

Depression is among the most prevalent mental health disorders, affecting over 300 million people of all ages worldwide, and has become the leading cause of disability [1]. To reduce the burden of the disease, alleviate symptoms, and prevent or mitigate serious consequences, professional treatment is indicated [2–4]. When left untreated, depression tends to become chronic, is more likely to be accompanied by comorbidities [5], and incurs significant costs [6]. However, despite the presence of an objective need, characterized by a clinically relevant diagnosis with available effective treatment options [2–4], recognition and treatment rates for depression remain low. Consequently, only 20% of participants with major depressive disorder in high-income countries received minimally adequate treatment [7]. Analyses from the German Health Interview and Examination Survey for Adults (DEGS) reveal that only 34.6% of individuals who met the diagnostic criteria for major depression in the past 12 months used specialized mental health services (MHS) [8]. Moreover, individuals with depressive disorders often seek professional help only after a considerable delay [9, 10].

There is already substantial knowledge about the possible reasons why the delayed or absent treatment of depression remains a significant issue. In addition to various structural barriers (e.g., availability of therapy slots) and attitudinal barriers (e.g., stigma) [11], the failure to recognize depression symptoms as such also plays a significant role. Vignette studies have shown that the correct diagnosis rate has only improved by 20% (to 46.3%) over the past 30 years and remains relatively low [12], despite increased efforts to enhance mental health literacy [13, 14]. Recognizing one’s own symptoms of depression is a critical first step in the seeking mental health care process. Without this self-awareness, the initiation of appropriate help is unlikely to occur, as no perceived need can be formed [15]. When examining potential barriers to seeking mental health treatment, the most frequently cited reason for not initiating care was a low perceived need, with women and younger individuals being more likely to recognize a need for treatment [11].

In addition to the absence of perceived need as a barrier to seeking treatment, recent research has highlighted the importance of recognizing this need in initiating care. Thoits [16] found that perceived need is a stronger predictor of the utilization of psychotherapy and pharmacotherapy among individuals with various mental disorders compared to clinical need. Specifically, two-thirds of all respondents who reported a subjective need also received professional treatment [16], which is a significantly higher percentage compared to the met needs reported in studies based on objective need [7, 8]. Furthermore, the results revealed perceived need for treatment increased with the presence and seriousness of current mental disorder: individuals with more serious current disorders were more likely to perceive a need for treatment than those with less serious current disorders, while perceived need was lowest among individuals who had recovered from a prior disorder [16].

Despite this relevance, a systematic review indicated that the subjective need of individuals with major depressive disorder has been insufficiently studied as a factor influencing help-seeking behavior, compared to other factors such as symptom severity or gender [17]. Up to date, the quantification of an unmet need is assessed based on the objectively diagnosed disorder. Moreover, in the treatment guidelines, the subjective need of affected individuals also play a secondary role, with the objective need being the primary factor determining treatment recommendations [2–4].

Reasons for relying on objective criteria when making treatment recommendations and calculating treatment rates include the need for standardization and objectivity. Validated diagnostic interviews [18–21] based on the criteria of the International Classification of Diseases, 10th Revision (ICD-10), or the Diagnostic and Statistical Manual of Mental Disorders, Fifth Edition (DSM-5), provide objective diagnoses and ensure consistency in treatment recommendations across different clinical settings and practitioners. They also provide a reliable assessment of disease severity, which is then used to guide treatment recommendations within clinical guidelines. In Germany, the National Treatment Guideline for Unipolar Depression recommends psychotherapy or pharmacotherapy for mild depression lasting more than two weeks, as well as for moderate depression. For severe depression, a combination of pharmacotherapy and psychotherapy is advised [4]. While these recommendations are based on clinical assessments, relying solely on self-reports to determine treatment need may lead to underestimation, as individuals might underreport symptoms or fail to recognize their need due to attitudinal barriers such as stigma [22, 23]. Objective measures, therefore, help ensure that individuals with similar diagnoses receive comparable care.

However, the role of subjective need in explaining treatment gaps or delays among individuals with depressive symptoms should not be overlooked. Both objective and subjective need are critical factors influencing treatment decisions. Guideline recommendations may be inappropriate for certain individuals, even when an objective need is present, particularly in cases where there is a discrepancy between objective and subjective need. To our knowledge, no study has yet examined individuals with depressive symptoms or disorders in which such an incongruence exists.

Therefore, this study aimed to examine guideline-compliant use and non-use of professional mental health services among individuals with depressive symptoms in relation to congruence or incongruence between diagnosis-based objective need and subjective need. Quantitative data were first used to quantify need congruent and incongruent patterns of mental health service use. Qualitative interviews were then conducted to explore the conditions and reasons underlying guideline-compliant use or non-use of pharmacotherapy and/or psychotherapy, particularly when objective and subjective need did not align.

## Methods

### Study design

**Fig. 1:**
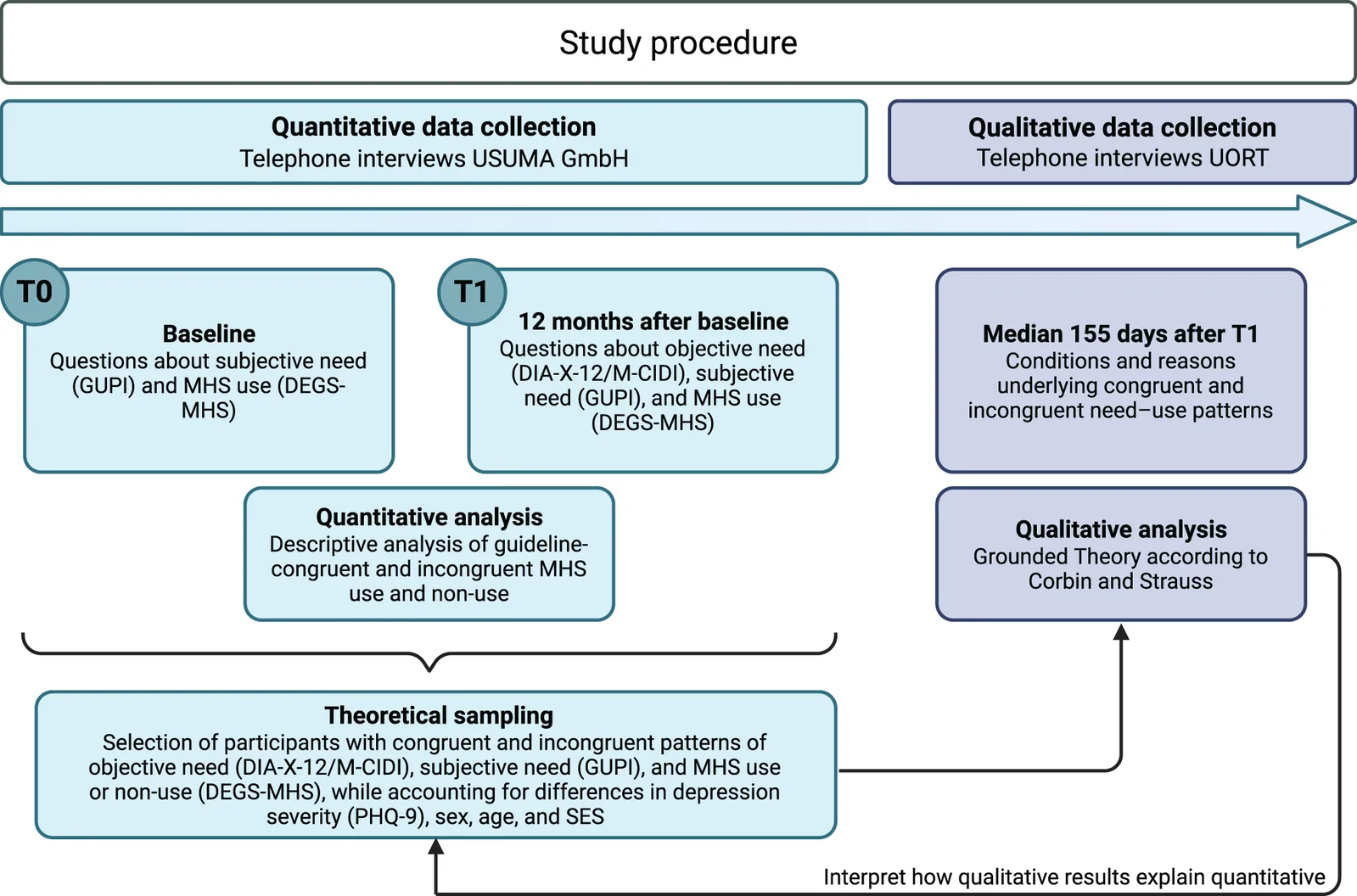
Study procedure and data collection steps within the sequential explanatory mixed-methods design. Abbreviations: DEGS-MHS: German Health Interview and Examination Survey for Adults - Mental Health Module; DIA-X-12/M-CIDI: Munich - Composite International Diagnostic Interview; GUPI: General-practice Users’ Perceived-need Inventory; MHS: mental health services; PHQ-9: Patient Health Questionnaire-9; UORT: University of Oldenburg research team

The quantitative and qualitative data were integrated using a sequential explanatory mixed-methods design [24]. The quantitative arm was based on a representative nationwide community sample recruited from the general population via telephone numbers and subsequently restricted to individuals with at least mild depressive symptoms. Participants were recruited irrespective of their objective need, subjective need, or mental health service use at baseline. Quantitative data were collected and analyzed at two time points, baseline (T0) and 12 months later (T1), within the nationwide study titled “Influence of established and subjectively perceived as well as evaluated individual characteristics on the utilization of mental health services among individuals with depressive disorders” [25]. Subsequently, qualitative interviews with a theoretically selected sub-sample were conducted to gain a more comprehensive understanding of the utilization behavior of individuals with incongruent needs. The qualitative findings were then used to explain the quantitative results, thereby enriching the overall interpretation (see Fig. 1).

The study was funded by the German Research Foundation and approved by the ethics committee of the Carl von Ossietzky University (ID: 2018–090). To ensure data security, the study was conducted in full compliance with General Data Protection Regulation. The design of the questionnaire, the analysis of the quantitative data, and the subsequent conduct and analysis of the qualitative interviews were carried out by the University of Oldenburg Research Team (UORT).

### Inclusion criteria and recruitment

Inclusion criteria at T0 were an age of 18 years or older and a score of five or more in the Patient Health Questionnaire-9 (PHQ-9) [26], reflecting at least mild depressive symptomatology. Participants were interviewed via computer-assisted telephone interviews. To guarantee representativeness, the sample was generated from landline and mobile numbers according to the Association of German Market and Social Research Agencies’ dual-frame approach [27]. Telephone numbers from a selection framework were processed using an adaptive generation method [28] and were selected randomly. Transferable numbers were contacted up to ten times on different days of the week and at various times of the day to improve contact success.

### Data collection

#### Quantitative data

For reasons of feasibility, the quantitative data was collected by the independent social research institute USUMA GmbH between April and November 2020 (T0). After the questionnaire was created by the UORT and programmed by USUMA GmbH, a technical review, an interviewer training, as well as a pretest phase followed. The UORT developed training materials and conducted both on-site and online training sessions for the interviewers and supervisors from USUMA GmbH prior to each data collection wave. Before conducting the first telephone interview, participants’ oral informed consent was obtained and documented.

The required quantitative sample size for this nationwide study was 925 participants, as calculated and reported in the study protocol [25]. To achieve this sample size at T0, USUMA GmbH employed 58 interviewers and conducted 6,613 screening interviews. Of these, 674 participants refused to participate after the interviewers explained the rationale of the study, and 5,014 scored below five on the PHQ-9 [26] and therefore did not fulfil the inclusion criterion.

Before the second interview, participants were provided with answer sheets from the UORT to respond to selected questions in the upcoming diagnostic interview, as well as detailed information about the potential qualitative interviews. Twelve months after their first interview (T1), participants were contacted and interviewed again. At T1, USUMA GmbH deployed four interviewers with the necessary qualifications for conducting diagnostic interviews. At the end of the T1 interview, respondents were informed about the possibility of participating in a follow-up qualitative interview with the UORT and were given the opportunity to either consent or decline. The consent included the transmission of contact details (email and/or residential address) to the UORT. Further details on the survey method are available in the study protocol [25].

#### Qualitative data

The interviews were conducted by a 36-year-old female researcher (AKR) with a professional background in nursing and academic training in Public Health and Epidemiology. The interviewer was not known to the participants prior to the study and had no professional relationship with them. Participants eligible for a qualitative interview were contacted by the UORT, and written informed consent was obtained prior to conducting the interviews. The interviews focused on four key themes: (1) validation of selected items from the quantitative survey, (2) determinants of (non-)utilization of mental health services, (3) perceptions of needs-based care, and (4) experiences of stigma and related expectations toward treatment and services (see Supplementary file 1). A semi-structured format was used to ensure systematic coverage of these topics while allowing flexibility to explore themes arising during the interviews. Adjustments to the interview guide were made iteratively in accordance with the principles of the grounded theory approach [29].

The selection of participants for the qualitative interviews was based on the quantitative data according to the criterion of theoretical sampling [29]: Initially, individuals with a mismatch between objective and subjective treatment need were identified (Table 1). Only those with moderate to severe depression diagnoses were included to ensure homogeneity in objective treatment need. Within the incongruent groups, participants were selected based on their use or non-use of guideline-compliant MHS between T0 and T1 (inpatient/outpatient psychotherapy or pharmacotherapy). This process resulted in four main subgroups (Table 1). Due to the low number of cases in the groups “objective need/no subjective need” and the high heterogeneity observed in the initial interviews within the groups “no objective need/subjective need,” we focused our sampling on the latter. This decision was made in line with the logic of theoretical sampling [29], as further interviews were conducted to explore and refine categories developed through the ongoing analysis [29]. To further contrast the findings with cases in which there is complete congruence between objective and subjective need and MHS utilization, we also decided to conduct interviews in these groups as well (Table 2), resulting in a total sample size of 23 interviews. When selecting participants across the six groups, which reflected different combinations of objective need, subjective need, and MHS use or non-use (Tables 1 and 2), care was taken to account for differences in their levels of depression [26, 30], gender, age, and socioeconomoc status index (SES) [31]. Theoretical sampling also allowed for ongoing adjustments to the interview guide to explore concepts identified and further developed through the ongoing analysis [29]. Interviews were conducted via telephone due to the Covid-19 pandemic.

**Table 1:** Overview of identified participants and conducted qualitative interviews among individuals with incongruent treatment needs

	New utilization of guideline-compliant MHS	No utilization of guideline-compliant MHS
Incongruence of need	Eligible participants/conducted interviews	Eligible participants/conducted interviews
No objective need, subjective need	19/12^a^	43/7
Objective need, no subjective need	1/1	6/4

^a^Five of the 12 interviews had to be excluded due to the presence of comorbidities not screened for in advance or due to reported MHS utilization related to a sleep disorder not associated with depression

MHS: mental health services

**Table 2:** Overview of identified participants and conducted qualitative interviews among individuals with complete congruence between treatment objective and subjective need and utilization of guideline-compliant mental health services

Congruence of need	Congruent presence of both treatment need and guideline-compliant MHS utilization	Congruent absence of both treatment need and MHS utilization
Eligible participants/conducted interviews	10/2	45/2

MHS: mental health services

The median interval between T1 and the subsequent qualitative interview was 155 days. All interviews were transcribed verbatim by a commissioned transcription service and anonymized by the UORT. The quotations shown were translated verbatim from German into English by the UORT.

### Measures

In the following sections, we explain only the instruments relevant to answering the questions posed in this paper (for further information see Supplementary file 2: Table S1). A complete list of the instruments used to collect data can be found in the study protocol [25].

**Subjective need** for guideline-compliant treatment was recorded at T1 using items two (pharmacotherapy) and three (counselling/psychotherapy) from an adapted version of the General-practice Users’ Perceived-need Inventory (GUPI [32]). This adaptation, piloted in a mixed-methods study of depressive patients (unpublished results; for further information, see [25]), evaluates subjective need regardless of current treatment status. Participants respond to five items in total with one of the following options: “I would like to use this offer of help,” “I do not want to use this offer of help,” “I already use this offer of help and I have also wanted to use it,” and “I am already using this offer of help, but I did not want to use it.” For this analysis, responses were dichotomized by combining the first and third response options (indicating perceived need for care) and the second and fourth response options (indicating no perceived need for care). Pharmacotherapy and counselling/psychotherapy needs were then pooled into one variable for analysis.

An **objective need** for treatment according to the National Clinical Practice Guideline for Unipolar Depression [4] has been assessed using the Munich - Composite International Diagnostic Interview (DIA-X-12/M-CIDI [33, 34]) at T1. The computer-aided interview enables the recording of 12-month diagnoses according to ICD-10 based on algorithms. Only sections E (depressive disorders and dysthymic disorders) and F (mania and bipolar affective disorders) were surveyed. An objective need was identified if respondents had a single diagnosis of mild, moderate, or severe depression (F32.0–F32.2 and F33.0–F33.2). Those without any diagnosis according to the DIA-X [33, 34] were considered not to have an objective need for treatment.

The **depression symptomatology** experienced over the past two weeks was assessed using PHQ-9 scores at both time points [26, 35]. Each of the nine items aligns with one of the DSM-IV [36] criteria for diagnosing major depression. Participants rated their agreement with each item (e.g., “Little interest or pleasure in doing things”) on a four-point Likert scale, ranging from 0 (“not at all”) to 3 (“nearly every day”), resulting in a total score that can range from 0 to 27. The internal consistency of the questionnaire at T1, with a Cronbach’s alpha of 0.77 for depressive symptomatology, is slightly lower than the 0.89 reported in validation studies [30] but remains within an acceptable range [37].

**Mental health care utilization** was surveyed based on the procedure in the DEGS mental health module [38]. For the analyses in this study, only guideline-specific mental health care services [4] were considered, reflecting inpatient and outpatient psychotherapeutic treatment options (psychiatric, psychotherapeutic or psychosomatic clinics, departments, day-care hospitals, and outpatient clinics, psychosomatic rehabilitation, established psychotherapists and psychiatrists/neurologists) as well as prescription pharmacotherapy. Lifetime, 12-month, and current utilization of mental health care services was assessed at T0, while 12-month and current utilization was reassessed at T1.

Subjects were screened for the three **comorbidities:** generalized anxiety disorder (Generalized Anxiety Disorder Scale-7 [39, 40]), alcohol use disorder (Alcohol Use Disorders Identification Test - Consumption [41–43]), and somatic symptom disorder (Patient Health Questionnaire 15 [26, 35] and Somatic Symptom Disorder Scale-12 [44]) at T1. Using the expectation-maximization algorithm [45], missing values from the screening instruments were replaced if no more than 25% of the values per subject and instrument were missing (missing values per item ranged from 0.2% to 7.4%).

**The socioeconomic status index (SES)** was calculated based on the data on education, job situation, and net equivalence income (computed from the size of the household and income/financial situation of the respondent) [31] at T0. If missing data occurred in one of the three individual dimensions, we imputed the mean score of the remaining two dimensions. Missing data in more than one dimension meant that the SES index could not be calculated and was therefore missing. Further sociodemographic characteristics reported are age, sex, family status, and migration background (self or at least one parent not born with German citizenship [46]).

### Data analysis

Data were analyzed using a mixed-methods approach with a sequential design, combining quantitative and qualitative techniques to provide a comprehensive understanding of the research questions. Individuals with diagnoses other than depression (e.g., hypomania or bipolar disorder) were excluded from both analyses to ensure that our results specifically reflect depression diagnoses.

#### Analysis of quantitative data

To determine the proportion of respondents whose MHS use corresponded to their objective need, we first calculated how many respondents reported having received mental health care between T0 and T1, which was then classified as guideline-compliant or non-guideline-compliant according to the national guideline [4]. It was irrelevant whether the participants had newly initiated this utilization between T0 and T1 or whether it was a continuation of an ongoing utilization. In contrast, we also assessed the ratio of individuals who used services incongruently with their objective or subjective need. Furthermore, we reported the proportion of participants who used MHS despite having no confirmed diagnosis according to the diagnostic interview [33, 34]. Data were analyzed using SPSS (Version 26) [47], and participants with missing values for subjective need were excluded from the analysis.

#### Analysis of qualitative data

The analysis of the second research question was conducted in accordance with Grounded Theory by Corbin and Strauss [29]. The core of this method is the continuous comparison of the interview material through a three-stage coding process. Open coding was carried out simultaneously by two researchers (AKR and EB). Initially, the most important text passages were labelled with codes, which were then grouped into categories in the next step. After the codes were created and after the categories were formed, each interview was discussed until a consensus was reached. During this process, the text passages, codes, and categories were repeatedly compared with those from previous interviews, and memos were used to document the properties and dimensions of the categories and potential relationships between categories. The next two coding steps were then carried out collaboratively through discussion (AKR and EB). For axial coding, relationships between the categories were visualized using coding paradigms. These paradigms were then compared with each other again, and if necessary, categories were modified or supplemented during this analysis step (circular process). Finally, in the selective coding phase, a typology of the subjective need was established. The qualitative analysis was performed with MAXQDA 2020 [48].

### Results of quantitative analysis

Of the initial 925 target individuals, 788 remained available for follow-up at T1. Reasons for dropout included refusal to participate, death, relocation, or medical conditions, as reported by the target individuals themselves or by contact persons. During T1, 257 additional cases were lost due to refusals, discontinuations, or failed contact attempts, resulting in 531 completed interviews and a dropout rate of 42.6%. The average interview duration was 39 minutes at T0 and 55 minutes at T1.

To address the first research question, participants with dysthymia (*n* = 47), double depression (*n* = 19), organic affective disorder (*n* = 1), hypomania (*n* = 2), or bipolar disorder (*n* = 2) were excluded, so that only individuals with a diagnosis of mild, moderate, or severe depression—or no diagnosis at all—were included in the analysis. Additionally, four individuals were excluded due to missing values on variables related to subjective need. The final analysis sample comprised 456 participants, with descriptive statistics presented in Table 3.

**Table 3:** Characteristics of the quantitative and qualitative study samples

	**Quantitative Sample** (*n* = 456)	Qualitative Sample (*n* = 23)
Sex, n (%)		
Male	206 (45.2)	11 (47.8)
Female	249 (54.6)	12 (52.2)
Diverse	1 (0.2)	-
Age in years, mean (SD)	54.2 (17.8)	51.7 (18.5)
Socio economic status index, n (%)		
Low	37 (8.1)	3 (13.0)
Middle	200 (45.2)	11 (47.8)
High	215 (47.1)	9 (39.1)
Missing	4 (0.9)	-
Migrant status, n (%)		
Yes	93 (20.4)	8 (34.8)
No	363 (79.6)	15 (65.2)
PHQ-9 Score T0, mean (SD)	7.7 (2.9)	8.0 (2.7)
PHQ-9 Score T1, mean (SD)	6.7 (4.0)	6.0 (2.5)
Objective need according to DIA-X^1^		
Yes	83 (18.2)	7 (30.4)
No	373 (81.8)	16 (69.6)
Risk of comorbidities T1		
Yes	205 (44.8)	0 (0.0)
No	245 (53.7)	23 (100.0)
Missing	7 (1.5)	-
Subjective need for Pharmacotherapy and/or counselling/psychotherapy T1		
Yes	287 (62.9)	16 (69.6)
No	169 (37.1)	7 (30.4)
Utilization of mental health services between T0 and T1, n (%)^2, 3^		
None	334 (73.2)	14 (60.9)
Inpatient	13 (2.9)	2 (8.7)
Of which newly initiated	8 (61.5)	2 (100)
Outpatient	81 (17.8)	8 (34.8)
Of which newly initiated	43 (53.1)	8 (100)
Medical	70 (15.4)	0 (0)
Of which newly initiated	17 (24.3)	0 (100)

Reported characteristics refer to time point T0 unless otherwise specified

^1^“Yes” includes mild, moderate, and severe depression diagnoses for the quantitative sample, and moderate and severe depression diagnoses for the qualitative sample

^2^The definition of mental health service utilization differs slightly between the quantitative and qualitative samples. In the qualitative analysis, only participants with either new or no utilization were considered

^3^Multiple responses possible

Of all participants with a mild or moderate depression diagnosis, 19% received psychotherapy or pharmacotherapy within the 12 months between T0 and T1 and were thus guideline-compliant (Table 4). A lack of treatment due to non-utilization was observed in 71.4% of the participants. The remaining 9.5% received a combination of psychotherapy and pharmacotherapy, which does not align with guideline recommendations. In contrast, combination therapy is indicated for severe depression, and 26.8% of respondents with a corresponding diagnosis reported receiving such treatment. However, a total of 73.2% of them received either no treatment (31.7%) or only psychotherapy or pharmacotherapy (41.5%). Among participants without a depression diagnosis, 22.5% used one or more services, contrary to guideline recommendations. Notably, only two out of 83 individuals with a depression diagnosis used MHS without reporting a subjective need (2.4%), and five used a treatment type that did not correspond to their reported need for psychotherapy and/or pharmacotherapy (6.0%). Furthermore, 219 out of 290 individuals without a depression diagnosis (75.5%) reported one or two subjective need (*n* = 46 for two needs; Table 4).

**Table 4:** Utilization of professional mental health care services by participants according to their subjective and objective need status between T0 and T1

	Individuals with a mild (*n* = 15) or moderate (*n* = 27) depression diagnosis	Individuals with a severe depression diagnosis (*n* = 41)	Individuals without a diagnosis (*n* = 373)
PHQ-9 Score T1, median (IQR; min, max)	8 (6.0; 0, 15)	9 (7.5; 2, 27)	5 (4.0; 0, 19)
**No utilization**, n (%)	30 (71.4)	13 (31.7)	289 (77.5)
No subjective need	8 (26.7)	5 (38.5)	150 (51.9)
Subjective need for counselling/psychotherapy	21 (70.0)	6 (46.2)	116 (40.1)
Subjective need for pharmacotherapy	0 (0.0)	1 (7.7)	9 (3.1)
Subjective need for counselling/psychotherapy and pharmacotherapy	1 (3.3)	1 (7.7)	14 (4.8)
**Utilization of psychotherapy**, n (%)	5 (11.9)	8 (19.5)	36 (9.7)
No subjective need	0 (0.0)	1 (12.5)	2 (5.6)
Subjective need for counselling/psychotherapy	5 (100.0)	7 (87.5)	30 (83.3)
Subjective need for pharmacotherapy	0 (0.0)	0 (0.0)	0 (0.0)
Subjective need for counselling/psychotherapy and pharmacotherapy	0 (0.0)	0 (0.0)	4 (11.1)
**Utilization of pharmacotherapy**, n (%)	3 (7.1)	9 (22.0)	25 (6.7)
No subjective need	0 (0.0)	0 (0.0)	1 (4.0)
Subjective need for counselling/psychotherapy	1 (33.3)	0 (0.0)	5 (20.0)
Subjective need for pharmacotherapy	2 (66.7)	3 (33.3)	6 (24.0)
Subjective need for counselling/psychotherapy and pharmacotherapy	0 (0.0)	6 (66.7)	13 (52.0)
**Utilization of psychotherapy and pharmacotherapy**, n (%)	4 (9.5)	11 (26.8)	23 (6.2)
No subjective need	1 (25.0)	0 (0.0)	1 (4.3)
Subjective need for counselling/psychotherapy	0 (0.0)	3 (27.3)	6 (26.1)
Subjective need for pharmacotherapy	1 (25.0)	0 (0.0)	1 (4.3)
Subjective need for counselling/psychotherapy and pharmacotherapy	2 (50.0)	8 (72.7)	15 (65.2)

### Results of qualitative analysis

The aim of the qualitative analysis was primarily to examine the utilization behavior of participants with incongruent needs more closely. To obtain a comprehensive picture, the decision was made during the course of the evaluation to include congruent cases in the analysis as well. The quantitative data already showed that utilization without subjective need or with a divergent need was rare. In contrast, non-utilization despite objective need was observed in more than half of all respondents with a depression diagnosis (Table 4).

The qualitative sample comprised 23 participants, with the characteristics of the sample depicted in Table 3. The average interview duration was 36 minutes. A total of 57 coding paradigms were developed, utilizing 371 main categories.

During the qualitative analysis, based on Grounded Theory [29], the pattern identified in the quantitative analysis became more pronounced, emphasizing that subjective need play a central role in explaining respondents’ utilization behavior. Furthermore, it became evident that a dichotomous distinction between the presence and absence of subjective treatment need does not adequately capture the reality of the individuals affected.

Therefore, within the framework of a typology of subjective need, six different types with a total of seven sub-types were defined (Table 5). The typology was based on the following characteristics: expression of subjective need, contact with the care system, and coping/utilization of mental health care services. The types and sub-types should be understood as analytically distinguishable patterns of need and service use at a given point in time rather than as fixed categories, as individuals may shift between them when their symptoms, perceived need, or service use change.

**Table 5:** Typology of subjective need in individuals with depressive symptoms

	**Type 1:** Barriers outweigh subjective need (*n* = 2)	**Type 2:** Subjective need outweighs barriers (*n* = 2)	**Type 3.1:** Subjective need met through treatment experience (*n* = 5)	**Type 3.2:** Subjective need met through treatment experience and changes in life circumstances (*n* = 3)	Type 4.1*: Subjective need dependent on professional assessment –>Subjective need is questioned (*n* = 1)
Expression of subjective need					
Type of need	Outpatient psychotherapy	Outpatient psychotherapy	No subjective need	No subjective need	Outpatient psychotherapy
Degree of expression	Moderate	Strong	(Preliminarily) met through utilization in the recent or distant past	Met through utilization in the recent past and/or changes in life circumstances	Moderate
Temporal dimension	Duration of psychological complaints varies greatly	Duration of suffering period varies	Long suffering period before utilization	No prolonged period of suffering before utilization	Long suffering period without utilization
Trigger	Symptoms triggered by an external stress situation	Increasing stress situation or symptomatology	Symptoms triggered by internal and/or external stress situations	Symptoms triggered by an external stress situation	Symptoms triggered by an external stress situation
Contact with the care system					
Previous treatment experience	Inpatient or outpatient psychotherapy and pharmacotherapy	None	Varies (reported one or more: inpatient/ outpatient psychotherapy, pharmacotherapy)	Varies (reported none, one or more: inpatient/ outpatient psychotherapy, pharmacotherapy)	None
Type of relationship with healthcare providers	Trusting	Trusting	Trusting	Trusting or difficulty assuming the patient role	Strained
Social support during initiation of treatment	None	Emotional	Active or emotional	Active, emotional or none	Emotional
Barriers/facilitating factors	Structural, attitudinal and illness-related barriers hinder utilization according to subjective need	The realization of service utilization is perceived as a ‘matter of luck	Structural and attitudinal barriers are mitigated by facilitating factors	Structural and attitudinal barriers are mitigated by facilitating factors or resolved need resulting from changes in life circumstances	Structural and attitudinal barriers impede utilization aligned with subjective need
Coping/utilization					
Coping with psychological complaints	In cases of solely subjective need, development of self-directed coping strategies in proximity to care; with additional objective need, guideline-compliant coping includes regular psychiatric appointments associated with long-term pharmacotherapy No use of (further) professional services in the past 12 months	Initiation of professional support before the onset of symptom-related incapacitation Development of coping strategies through outpatient psychotherapy in the past 12 months or the imminent start of outpatient psychotherapy	Development of coping strategies for managing symptom through professional support or sense of self-efficacy gained through a one-time visit to a psychiatric outpatient clinic Completion of outpatient psychotherapy/counselling in the past 12 months; in one case, ongoing regular psychiatric appointments associated with long-term pharmacotherapy	Development of coping strategies for managing symptoms through professional support or premature discontinuation of treatment due to prolonged wait times, resulting in the resolution of the stress situation Completion of inpatient and/or outpatient psychotherapy in the past 12 months or in the near future	Development of self-directed coping strategies to address need No use of professional services in the past 12 months
Objective need	n = 1	n = 0	n = 3	n = 0	n = 0
	**Type 4.2**: Subjective need dependent on professional assessment –>Subjective need is brought to awareness (n = 1)	Type 5.1*: Hypothetical subjective need (n = 2)	Type 5.2*: No subjective need for ‘real’ psychological treatment (n = 2)	Type 5.3*: No subjective need, as the level of distress is not significant enough (n = 3)	**Type 6**: Uncertainty about further handling of subjective need (n = 2)
Expression of subjective need					
Type of need	Pharmacotherapy	Pharmacotherapy	Low-threshold mental health care	No subjective need	Outpatient psychotherapy
Degree of expression	Strong and outcome-oriented	No subjective need	Moderate, but distancing from the need for professional support	Not applicable	Moderate
Temporal dimension	Long suffering period with a delayed association of symptoms with potential mental illness	Not applicable	Temporary suffering (associated with an external stress situation)	Duration of suffering period varies and is currently overcome	Long suffering period before or without utilization
Trigger	Uncertainty about causes despite numerous internal and external stress situations	Hypothetical triggers limited to external stress situations	Symptoms triggered by an external stress situation	Symptoms triggered by internal and/or external stress situations	Symptoms triggered by internal and/or external stress situations
Contact with the care system					
Previous treatment experience	Negative long-past treatment experience; emergency care opens a new access to the care system	None or long-past treatment experience	Formative experiences as a healthcare provider or service user	None or long-past treatment experience	Current or long-past treatment experience
Type of relationship with healthcare providers	Supportive	Hypothetical supportive	Hypothetical supportive	Hypothetical supportive	Suspicious or trusting
Social support during the initiation of treatment	Active and emotional	Not applicable	None	Not applicable	None or emotional
Barriers/facilitating factors	Objective symptoms/ diagnosis necessitates emergency care and foster the development of a subjective need	Attitudinal barriers hinder engagement with potential subjective need	Attitudinal barriers and/or competing life responsibilities hinder the utilization of professional services	Attitudinal as well as structural barriers and/or competing life responsibilities prevent use of services as long as the burdens persist	Attitudinal and structural barriers contribute to uncertainty in handling subjective need
Coping/utilization					
Coping with psychological complaints	Symptom repression until emergency care Pharmacotherapy therapy and development of a subjective need for psychotherapy, along with the development of self-directed coping strategies for symptom management in the past 12 months	Relativization of psychological symptoms and distancing from potential mental illness as a coping strategy No use of professional services in the past 12 months	Relativization of psychological symptoms Use of coaching and low-threshold services as substitutes for psychological treatment No use of professional services in the past 12 months	Relativization of psychological symptoms Development of self-directed coping strategies, with partial additional support from the social environment, for symptom management No use of professional services in the past 12 months	Development of coping strategies through inpatient and outpatient psychotherapy, or relativization of psychological symptoms and use of low-threshold services in the past 12 months
Objective need	n = 1	n = 0	n = 1	n = 1	n = 0

^*^Indicates types in which participants did not use professional mental health care

#### Type 1: Barriers outweigh subjective need (*n* = 2)

Participants in this category experience a moderate subjective need for outpatient psychotherapy. However, structural, attitudinal, and illness-related barriers prevent them from using professional services according to their need:

*I2 “(sighs) Well, I have quite a bit of chaos in my life, for personal reasons, so I hardly ever get around to it [initiating psychotherapy], and there is always something more important or more urgent that needs to be done.”*

*I1 “Yes. The recognized psychotherapists, you know the names, you get an appointment in a quarter of a year or so, that’s no use to me.”*

Instead, they rely on self-directed coping strategies, partly developed in proximity to available care. Furthermore, the presence of an additional objective need is associated with the reporting of long-term pharmacotherapy. The use of the latter is considered necessary but undesirable, illustrating how barriers can not only overshadow subjective necessity but also lead to the continuation of less time-consuming treatment options (pharmacotherapy) against the patient’s wishes. The respondents describe their relationship with their current care providers as trusting. Although these providers (general practitioner (GP), psychiatrist) are aware of the respondents’ psychological problems, they do not explicitly recommend psychotherapy. Moreover, participants do not receive social support regarding the initiation of care utilization.

#### Type 2: Subjective need outweighs barriers (*n* = 2)

For this group, a strong subjective need for outpatient psychotherapy drives service use despite the presence of barriers:

*I12 “Yes, definitely. So, I would/I’ve initiated this [psychotherapy] now and am not willing to back down anymore. Because the situation, the overall situation, I have certainly postponed for a very long time, and it hasn’t gotten any better. And since I can’t improve it on my own initiative, I will definitely seek help. And if it doesn’t work out with this therapist, then I’ll try to find another one with whom I get along better.”*

These individuals perceive service access as a “matter of luck” rather than a consistent aspect of healthcare provision, but are motivated to seek professional support before their symptoms lead to incapacitation. In this process, they are emotionally supported by their social environment. Additionally, they generally trust professionals involved in the help-seeking and treatment process, including healthcare providers and psychotherapists, but state that they have changed or would change providers if they feel misunderstood, not taken seriously, insufficiently supported, or if there is a lack of rapport:

*I11 “So ultimately, I did not feel particularly well supported and then left this general practitioner and found another doctor.”*

The ability to overcome barriers reflects the crucial role of subjective need in their help-seeking behavior. The duration of perceived symptoms varies and cannot, therefore, be considered a decisive criterion for the pronounced subjective need.

#### Type 3.1: Subjective need met through treatment experience (*n* = 5)

These participants addressed their subjective need after prolonged periods of suffering by utilizing guideline-compliant mental health care services in the recent or distant past, which helped them develop effective coping strategies. Other factors contributing to the stability of the respondents include long-term pharmacotherapy, a supportive social environment, the experience of not being alone with the illness (interaction with fellow patients), and the availability of effective treatment methods in case of a deterioration in health:

*I3 “So what helped a lot was the contact with other patients. I think that was the biggest factor that helped me, just the exchange with others who are going through similar experiences. To know that you’re not the only one going through this. The therapies themselves were, in my opinion, not the main determining factor, nor the medications themselves. It was more about this exchange with others.”*

While barriers were present, they were mitigated by facilitating factors during the prior treatment process. Although some participants still have an objective need for treatment based on their diagnosis, their subjective need is now partly or fully resolved, reducing the urgency for further service use:

*I4 “Well, for one thing, it was because I didn’t really have the feeling that it [initiating psychotherapy] was, as I said, so acutely urgent again.”*

In the event of a deterioration in mental health, renewed service utilization is considered. The trusting relationship with current or former healthcare providers supports this attitude.

#### Type 3.2: Subjective need met through treatment and life changes (*n* = 3)

This subgroup benefits not only from prior inpatient or outpatient psychotherapy but also from significant changes in their life circumstances, such as regained physical mobility or entry into a new job. Social support at the initiation of prior treatment ranged from active or emotional support to no support at all. Structural barriers to care were mitigated by facilitating factors such as supportive healthcare professionals, fortunate circumstances, and the timely flexibility of participants:

*I10 “So, I was really lucky because the new colleague [psychotherapist] had just started. And she maybe didn’t have such a client base yet. And accordingly, I was flexible in my schedule because I was unemployed at the time. So, from that perspective, I think it was just right for me.”*

As a result, they reported no current subjective need for psychotherapy, regardless of whether they are in the final phase of therapy, have already completed it, or have discontinued it. Coping strategies developed through professional support and changes in life circumstances that resolved the stressful situation further reduce the need for ongoing services. Whereas one participant in this group had difficulty assuming the patient role due to their professional background and preferred to seek support from their social environment in future situations of perceived need, the other two participants reported trusting relationships with their healthcare providers and expressed openness to utilizing professional MHS again.

#### Type 4.1: Subjective need dependent on professional assessment – questioned (*n* = 1)

The participant in this category exhibits a moderate subjective need for outpatient psychotherapy but relies heavily on professional assessment to validate this need. However, this need was dismissed by their GP in the past:

*I13 “No, that was very/ that was also very counterproductive because I felt that he [the GP] didn’t take it seriously or didn’t take my situation seriously. Exactly, that he/ that he downplayed it. He did give me the referral and also a list of phone numbers, but what he said otherwise was very counterproductive for me in that moment, and I still see it that way now.”*

As a result, strained relationships with the healthcare provider, along with attitudinal and structural barriers, hinder the initiation of outpatient psychotherapy, leading to no professional care being used in the past (12 months). Without the benefit of prior experiences with mental health care, the participant has developed personal coping strategies to address their subjective need, including maintaining contact and communication with close friends and family.

#### Type 4.2: Subjective need dependent on professional assessment – brought to awareness (*n* = 1)

This type includes an individual who initially suppressed symptoms until emergency care revealed both an objective and, initially, a subjective need for pharmacotherapy. Despite prolonged periods of suffering and numerous internal and external stressors, uncertainty about the causes of depression persists. Negative past treatment experiences may have unconsciously hindered the realization of subjective need. Emergency care provides a new entry point into psychiatric services, which is evaluated positively. The positive experience and improvement in condition through the use of pharmacotherapy make the utilization of outpatient psychotherapy conceivable:

*I14 “Yes, I think so too, and I will need that [psychological help]. And I didn’t have that before, but why should I go into therapy or seek help if I don’t need it? There are people who are much, much worse off than me and they are the ones who need help more. Where I then just say, they really need help. Now I have to say, unfortunately, I need it too.”*

The initiation of utilization is facilitated by a supportive social and medical environment.

#### Type 5.1: Hypothetical subjective need (*n* = 2)

Respondents in this category emphasizes that they have interpreted the question about their subjective need as hypothetical. In the event of a future need, they would prefer pharmacotherapy to numb negative emotions:

*I15 “But I expect that it will be numbed somehow, if you have some kind of, yeah, yeah, some kind of trauma, that it might be better—yeah, that it will just be numbed. For example, in the case of a death or something like that, for instance, that this, yeah, feeling becomes somewhat numbed. And over time, they say time, yeah, it helps. Then you would hope that with time it eases a bit.”*

Only external triggers for potential psychological symptoms, such as the death of a close person, are mentioned. However, all respondents exhibited at least mild depressive symptoms at T0. Attitudinal barriers (e.g., the belief that self-reflection does not require a therapist) prevent further engagement with their symptoms. Additionally, the symptoms are downplayed, leading to distancing from potential mental illness as a coping strategy. Consequently, no professional care services were utilized in the past 12 months. However, in the event that service utilization becomes necessary, all respondents expect support from potential healthcare providers.

#### Type 5.2: No subjective need for real treatment (*n* = 2)

This type includes participants who explicitly express a need for low-threshold services while emphasizing that they do not have severe or any psychological problems and therefore see no need for professional support. Symptoms are attributed to temporary external stressors. Support from the social environment for service utilization is absent, partly because participants do not, or only very sporadically, talk about their problems. Attitudinal barriers, the downplaying of their own symptoms, and/or competing life demands (e.g., work, care for children) prevent awareness of their own situation and, consequently, the utilization of professional mental health care. Instead, participants engage in coaching and low-threshold services to manage their daily lives without identifying themselves as mentally ill:

*I17 “[…] I haven’t sought psychological counselling but rather a general consultation, or more like a kind of coach, where I have the opportunity to ask questions. This is because it’s more about interpersonal matters than directly about me as a person.”*

*I18 “I would never have seen that as a depression or anything like that, so that one would … yeah, I just didn’t … for me, it was never … not that I would fundamentally reject it, but I just didn’t … I would have thought there are probably enough other ways to resolve it.”*

If they decide to seek professional care in the future, they expect support from their GP in initiating psychotherapy or pharmacotherapy.

#### Type 5.3: No subjective need due to low distress (*n* = 3)

Type 5.3 includes respondents who did not indicate any subjective need during the qualitative interview. The reasons for this vary and are based on attitudinal barriers, competing life demands, or the perception that the stressful situation has already passed:

*I20 “I’m always torn. Sometimes I think, okay, maybe it [psychotherapy] would help me, and somehow, at the same time, I have this kind of fear of it. I can’t even describe why. Maybe because something might come up that I’ve already buried deep down or something like that. I don’t know. I really don’t know.”*

However, this self-assessment partially contradicts the findings of the diagnostic interview conducted at T1 for the diagnosis of depression. Nevertheless, all participants reported being able to manage their daily lives well. To achieve this, they have developed their own coping strategies, become experts in managing their symptoms, and, in some cases, receive support from their social environment. The participants are characterized by either long-past or no treatment experiences and trust in a supportive healthcare system in the event that service utilization becomes necessary.

#### Type 6: Uncertainty about further handling of subjective need (*n* = 2)

Participants in this category experience prolonged periods of suffering without using care. However, one participant has already begun outpatient psychotherapy, which will soon be terminated by the therapist due to their transition to private practice and relocation to another city. Structural and attitudinal barriers, as well as, in one case, a distrustful attitude toward healthcare providers, contribute to uncertainty about how to address unresolved subjective need moving forward:

*I23 “I think that’s partly why I had trouble even thinking about psychological help for a long time. Because the thought was, okay, first, I’d be agreeing with them [mental health care professionals], and second, yeah, then you’re kind of labelled as someone who has a problem, right?”*

The management of psychological issues over the past 12 months involves the development of coping strategies through therapeutic support or the occasional utilization of low-threshold forms of care. However, these coping strategies appear insufficient as the psychological problems lead to prolonged illness-related absenteeism or difficulties in pursuing a professional career:

*I23 “I already have a hard time motivating myself, so I’d rather motivate myself for just one thing, like work, instead of a second thing like therapy. You know? That’s the thought.“*

Overall, respondents—particularly when discussing pharmacotherapy—expressed that they did not feel taken seriously when asked about their potential desire for treatment. They emphasized that no one wishes to be ill and require treatment, suggesting that the wording of questions assessing subjective need may require revision. However, among respondents with a subjective need for psychotherapy, this type of criticism did not arise when further inquiry was made regarding whether the utilization aligned with their personal wishes. In addition, the respondent’s subjective need persists until the utilization of MHS occurs, but not as a result of the “wait-and-see” strategy.

For most types and subtypes, any perceived subjective need primarily related to psychotherapy or low threshold MHS rather than pharmacotherapy, with the exception of types 4.2 and 5.1. Pharmacotherapy was generally viewed more critically and was often met with reservations, including fears of side effects or even past experiences with them, as well as a stigmatizing attitude toward medication.

*I11” […] I always wanted to have this clear view of myself and not somehow, yes, let myself be fogged up or something. That / I wanted to be very aware of myself, and that is why I / well, I blocked that [pharmacotherapy] from the outset. […] It was also clear to me that there would certainly be severe side effects, and I somehow did not want that either.”*

*I17 “Therefore, if one is already so mentally ill, I hope that I still have such a mind that I do not get into that, that I am still normal in that respect. Uh, so normal that I would not need / hopefully would not need such psychotropic medication, to put it that way, that I am still reasonably clear in my mind.”*

Interestingly, Type 5.1 showed the opposite pattern: Here, respondents described a hypothetical subjective need for pharmacotherapy, while psychotherapy was perceived as unhelpful and, in some cases, even stigmatizing.

Individuals with an objective need cannot be clearly assigned to a specific type but are distributed across various types without a discernible pattern (Table 5). However, it becomes evident that as subjective need increases, participants with objective need are also more likely to have utilized treatment in the past (Type 3.1) or to seek it in the present (Type 4.2). Non-utilization despite the presence of an objective need was always attributed to the absence of a subjective need (Types 3.1, 5.2, and 5.3). Within Type 1, non-utilization of psychotherapy results from attitudinal and structural barriers that outweigh the subjective need. However, long-term guideline-compliant pharmacotherapy ensures mental stability.

## Discussion

The results of this mixed-methods study indicate that the reasons for (non-)utilization of MHS in cases of incongruence between subjective and objective need are complex and multifaceted. The result, which was already evident in the quantitative data, has been confirmed by the qualitative data: service utilization in the absence of a subjective need is highly unlikely, and an objective need is not necessarily a reliable predictor of whether MHS will be utilized. Furthermore, the influence of subjective need on the help-seeking process cannot be interpreted in a binary manner (yes/no) but should always be considered in relation to the given context factors of those affected. Based on this, six types of subjective need were identified in the analysis of the qualitative data. The typology reveals that subjective need varies in intensity, duration, and resolution. While Types 1 and 2 emphasize the interplay between barriers and needs, Types 3 and 4 highlight the impact of treatment experiences on resolving or maintaining subjective need. Types 5 and 6 reflect how the absence or uncertainty of need shapes coping and utilization patterns.

The complexity of these patterns also supports previous critiques of purely rational-choice assumptions in help-seeking research [49]. Although respondents sometimes weighed perceived need, barriers, expected benefits, and prior treatment experiences, their decisions were also shaped by stigma, perceived legitimacy of need, trust in providers, social support, and emotional burden. The typology therefore suggests that MHS use and non-use cannot be fully explained by rational calculation, but must be understood in relation to the conditions under which subjective need is recognized, negotiated, and acted upon.

### The role of subjective need in the help-seeking process

Our findings confirm that subjective need plays a crucial role in the treatment process, acting both as a barrier and a facilitating factor, a concept that has also been demonstrated in previous studies [11, 16]. Moreover, MHS were primarily utilized when respondents perceived a subjective need, regardless of the presence of a clinical diagnosis. This aligns with prior research emphasizing that perceived need is central to the help-seeking process. For instance, Wix et al. [50] examined the role of subjective need in the initiation of treatment by GPs, highlighting its relevance alongside objective indicators in identifying patients with unmet mental health needs. Correspondingly, Thoits [51], drawing on self-labelling theory, demonstrated that individuals’ self-assessment of their mental health more reliably predicted treatment utilization than clinical indicators based solely on diagnosis. Her analysis further showed that when accurate self-labelling was considered, the proportion of seemingly inappropriate treatment use decreased significantly compared to estimations based solely on objective clinical indicators. In line with this, our findings show that a present subjective need, regardless of the presence of an objective need, was addressed only through utilization of professional services, not through a “wait-and-see” approach. The “watchful waiting” strategy recommended in the German guideline [4] for mild depression during the first 14 days appeared ineffective when subjective need was present. This underscores the clinical relevance of perceived need and the necessity of continuous monitoring to identify persistent depressive episodes with ongoing subjective burden. Accordingly, subjective need has been increasingly acknowledged as an important factor in national and international depression guidelines [2–4]. The three guidelines cited here emphasize that treatment decisions should be based not only on scientific evidence but also on patients’ individual needs and preferences. Shared decision-making (SDM) is explicitly recommended as the preferred approach to incorporate patients’ subjective need into mental health care planning. However, these recommendations remain at a general level and none of the guidelines distinguish the role of subjective need as a barrier or facilitator of help-seeking. Although possible themes or questions for SDM are mentioned, concrete guidance for managing incongruences between objective and subjective need is largely absent. Only one guideline [2] addresses the issue of incongruence, noting that discrepancies between clinical and subjective need may lead to treatment discontinuation. It therefore underscores the importance of also considering factors such as patients’ treatment history, potential barriers, and level of social support in the selection of appropriate treatment options. These considerations should also be addressed through an SDM process. It is the same guideline that includes individuals who self-report depressive symptoms without a formal diagnosis. In doing so, it explicitly considers subjective need not only as a treatment modifier but as a legitimate starting point for initiating care. In contrast, the two other guidelines [3, 4] restrict their applicability to patients with a confirmed diagnosis, thus limiting the relevance of subjective need in those contexts.

These findings call for a deeper conceptualization of “need” in the context of mental illness, as current assessments often rely on overly simplistic, dichotomous or three-level measures [50, 52], frequently based on single-item questions [50, 53]. To advance clinical applicability, future instruments should operationalize subjective need in terms of perceived treatment necessity rather than desire, a distinction especially relevant for pharmacological interventions, which were often stigmatized or associated with side effects in our study.

Our proposed typology of subjective need, comprising six types and seven subtypes, offers a first decision aid for professionals involved in the help-seeking and treatment process. For example, GPs may use it to better identify and support patients’ subjective need during initial help-seeking, while psychotherapists may use it to tailor treatment more closely to patients’ needs and expectations. By incorporating contextual factors such as treatment history, existing barriers, coping strategies, and social support, it enables more informed and individualized treatment decisions, particularly in cases of incongruence between clinical indicators and perceived need.

However, further research is required to validate the identified types in diverse populations, particularly among larger groups of individuals with objective but no subjective need. In parallel, a systematic review could clarify whether existing treatment guidelines address need incongruences in greater depth and offer more specific clinical recommendations.

### The role of objective need in the help-seeking process

The results of the quantitative analysis revealed that a notable proportion of participants with a confirmed diagnosis did not use MHS according to guidelines. Guidelines address the possibility of patients fundamentally refusing a recommended treatment in different ways. One guideline suggests offering the next level of recommendation [2], another advocates for accepting and documenting the refusal [3], and a third recommends accepting the refusal while providing continued support within the treatment process [4]. However, all three guidelines provide a clear recommendation for the utilization of professional mental health services in the presence of a depressive diagnosis, with variations depending on severity. Non-utilization can thus be defined as undertreatment. The qualitative data provided evidence that the absence of professional care services for individuals with a diagnosis of depression does not necessarily indicate inaction. For example, this may occur if coping strategies for managing symptoms were acquired during previous treatment experiences (Type 1) or if low-threshold assistance was utilized to alleviate the burden (Type 5.2). Overall, objective need played a secondary role in justifying service utilization among respondents. It was found to function as either a ‘driver’ or a ‘brake’—for instance, when worsening symptoms prompt GPs or family members to actively encourage utilization, or when symptoms become so severe that individuals cannot overcome access barriers without support.

### Strategies to increase need-congruent service utilization among those in need

The typology identified additional measures that may enhance service utilization. Across different subjective need types, structural barriers—such as caregiving responsibilities or the incompatibility of working hours with psychotherapy—were major obstacles to accessing care. To mitigate these barriers and increase the likelihood of service use, expanding online services could provide a flexible alternative. However, among the 264 respondents in our quantitative dataset who reported a subjective need for counselling or psychotherapy, only 15 (5.7%, results not shown) utilized internet-based services, including online programs provided by health insurance companies. Moreover, the vast majority of respondents in the qualitative interviews were unable to conceptualize online services, suggesting that both the dissemination and awareness of these offerings remain limited and require further expansion.

Beyond this, a lack of awareness regarding low-threshold services was evident among many respondents in qualitative interviews. Targeted referrals to these services could be particularly beneficial for individuals with subjective need only, as such referrals may help reduce perceived vulnerability and encourage early engagement with care. This is particularly significant because delayed utilization of services for depression is associated with an increased risk of chronicity [54]. Our study indicates, that prior treatment experience may contribute to better symptom management, as individuals with prior exposure to MHS might develop greater resilience or a better ability to interpret and endure their symptoms.

Furthermore, among individuals with an objective need, the threshold for seeking help was particularly high (Types 4.2 and 5.3) due to structural, behavioral, and illness-related barriers. In these cases, professional and/or social support was essential to facilitate access to care, as illness-related barriers could not be overcome by individuals alone. A persistent challenge remains in recognizing depressive symptoms and seeking help in a timely manner. Therefore, raising awareness of depressive symptoms and empowering patients to recognize when professional support is needed remain critical public health priorities. This is particularly relevant in depression, where symptoms and perceived burden may fluctuate and existing barriers may delay help-seeking. Digital tools that combine patient-reported outcome measure (PROM)-based monitoring with alert systems could support both patients and providers by continuously capturing subjective burden and helping to define individual thresholds for seeking professional support, either independently or within shared decision-making. Integrating patient-reported outcomes into routine care pathways would also align with the principles of value-based healthcare by supporting individualized, outcome-oriented care [55]. Importantly, these thresholds should be individually determined, as our typology shows that the point at which professional support is perceived as necessary depends on contextual factors such as existing coping strategies, prior treatment experiences, and the availability of social support. An integrated alert system could help patients monitor whether their individually defined threshold for service use has been reached. For instance, if a patient specifies that professional support should be sought when depressive symptoms prevent them from working or meeting friends on 10 out of 14 days, the system could provide timely feedback once this threshold is met and encourage help-seeking. Involving a GP in this evaluation process might further increase the likelihood of timely help-seeking and treatment initiation. The use of a digital monitoring could also be extended to individuals who do not yet meet the guideline-based diagnostic criteria for depression, in order to evaluate whether a watchful waiting approach is sufficient or whether further support is needed. The implementation of a comprehensive and standardized digital solution could therefore provide a promising and relatively cost-effective starting point for advancing toward a more need-sensitive and value-based system of care.

Moreover, increasing awareness of potential barriers could facilitate the timely utilization of MHS. Findings indicate that individuals without prior treatment experience, in particular, tend to assume that help will be readily available when needed. However, this expectation does not align with the reality of MHS provision in Germany. In 2018, the average time between initial contact and the start of psychotherapy was 20 weeks, which was longer than before the 2017 reform of the psychotherapy law, when the average wait time was 18 weeks [56]. Additionally, respondents with a hypothetical subjective need (Type 5.1) commonly expressed trust in their healthcare providers to assist them in seeking help. In contrast, those already in the process of help-seeking or receiving treatment consistently reported a desire for greater support from their providers.

In conclusion, it is noteworthy that all respondents were able to identify at least one potential point of contact to which they would turn in case of acute need.

## Limitations and perspectives

While our findings provide valuable insights, they should be interpreted in light of the following limitations. It should be noted that some participants interpreted the questions regarding subjective need hypothetically, which may have led to an overestimation of the proportion of individuals reporting a subjective need for treatment. However, this insight was only gained through the verification of need status in the qualitative interviews, and it cannot be ruled out that other instruments used to assess subjective need may also be partially understood in a hypothetical manner. Nevertheless, we chose the GUPI [32] as, to the best of our knowledge, only a few validated instruments are currently available for assessing subjective treatment need for mental disorders. As previously mentioned, there is a general lack of multidimensional validated assessment tools. For the development of future instruments, researchers should not only ensure a clear distinction between desire and need but also take measures to prevent questions from being interpreted hypothetically.

Accordingly, it should also be acknowledged that subjective need is dynamic and may change over time, which may not have been fully captured despite the prospective design of the study. Shorter monitoring intervals may be needed to assess subjective need more accurately, as perceived need may not remain continuously above a certain threshold but may fluctuate over days or weeks. This limitation further supports the need for regular monitoring of subjective need, as suggested above, for example through digital PROM-based assessment and individually defined alert thresholds.

Third, due to time constraints within the questionnaire, we only screened for three comorbidities commonly associated with depressive disorders. As a result, we had to exclude five qualitative interviews when it became evident during the interview process that participants had other comorbidities (e.g., eating disorders) or that service utilization was due to a sleep disorder unrelated to depression. To address this issue, we implemented an additional screening step in subsequent recruitment calls for the qualitative interviews, inquiring about other existing comorbidities in advance. This measure prevented further exclusions. Future studies should examine how comorbidities, which are common among individuals with depression [57], influence the treatment process, including recognition of patients’ perceived need for treatment.

Finally, the time interval between the T1 interview and the qualitative interview varied considerably. This variation resulted from the use of theoretical sampling, which aimed to include highly contrasting cases [29] and required the presence of an incongruence between subjective and objective need. Participants interviewed shortly after the T1 interview may have recalled their previous statements and experiences more accurately. At the same time, the variation in timing allowed for insights into how subjective need developed over time, both in individuals who used MHS and those who did not. To minimize variability in future research, larger sample sizes should be pursued to enable the selection of more comparable cases.

## Conclusion

This typology provides an initial framework for refining care approaches by enabling a more precise alignment with individuals’ perceived need through nuanced analysis. Our findings suggest that subjective need should play a greater role in decisions regarding service utilization, particularly in cases where there is an incongruence between diagnosis and perceived need. However, this requires the development of a validated instrument to accurately assess individuals’ subjective need and to support its systematic monitoring over time.

In addition to a validated instrument that allows for a more nuanced evaluation of subjective need beyond a dichotomous operationalization, a broader examination of the concept of need in the context of mental disorders is also necessary to realistically evaluate treatment necessity. Digital monitoring systems of depressive symptoms and subjective need, combined with individualized alert thresholds, could support this process by helping individuals and involved healthcare providers, such as GPs, psychiatrists, and psychotherapists, recognize changes in symptoms and subjective need over time and identify when professional support may be warranted. SDM could serve as a suitable framework for this purpose by enabling patients and healthcare providers, particularly GPs as initial points of contact, to jointly evaluate subjective need and make informed decisions about further treatment options.

Furthermore, treatment options should not be categorically ruled out for patients who lack an objective diagnosis but report a subjective need for help. The findings of this and previous studies [50, 51] indicate that patients are generally capable of accurately assessing their own need for support. In this context, subjective need may be defined as an awareness that a condition, which may fluctuate, is no longer manageable without professional help. Early engagement with MHS can promote the timely development of effective coping strategies and help prevent the progression to more severe clinical courses.

Instead of using objective need as the primary criterion for deciding on service utilization, it may be more beneficial to consider objective and subjective need in parallel, taking into account the diagnosis, existing barriers, prior treatment experiences, social support, and learned coping strategies. If subjective need were considered in a more nuanced way and its different types were given greater importance in treatment recommendations by healthcare providers, discussions on under- and over-treatment could be informed by new perspectives. Subjective perceptions of appropriate care may differ from clinically indicated care: treatment considered appropriate according to clinical guidelines may be perceived as insufficient by patients, while under- or over-treatment may nevertheless be perceived as appropriate, for example due to existing coping strategies or a pronounced subjective need and burden, respectively.

## Electronic supplementary material

Below is the link to the electronic supplementary material.

Supplementary material 1

Supplementary material 2

## Data Availability

The datasets generated and analysed during the current study are not publicly available to protect patient confidentiality. Selected variables are available from the corresponding author upon reasonable request.

## Abbreviations

DEGS-MHS: German Health Interview and Examination Survey for Adults-Mental Health Module
DFG: German Research Foundation
DIA-X/M-CIDI: Munich - Composite International Diagnostic Interview
DSM-5: Diagnostic and Statistical Manual of Mental Disorders, Fifth Edition
GP: General Practitioner
GUPI: General-practice Users’ Perceived-need Inventory
ICD-10: International Classification of Diseases, 10th Revision
MHS: Mental Health Services
PHQ-9: Patient Health Questionnaire-9
PROM: Patient-Reported Outcome Measure
SDM: Shared Decision-Making
SES: Socioeconomic Status Index
UORT: University of Oldenburg Research Team
