# Supplementary material for: No utilization without subjective need – a mixed-methods study on guideline-compliant mental health care for individuals with depressive symptoms
Source: BMC Health Serv Res. 2026 Sep 10;26:1261. doi: 10.1186/s12913-026-15562-3 (PMC13563709; doi:10.1186/s12913-026-15562-3)
Supplement: Supplementary file 1 [file 12913_2026_15562_MOESM1_ESM.docx]

Guiding questions for qualitative interviews

Some of the questions include alternative wordings. Depending on the respondent group (subjective need: yes/no; use of mental health services: yes/no), the appropriate formulation was selected in each case.

**1. Validation of Questionnaire Data**

- In the second telephone interview, you indicated that you [had/had not] used [type of service] in the past 12 months. Did we understand that correctly? Have there been any changes since then?
- You also indicated that the use of [type of service] [did/did not] correspond to your own preference. Did we understand that correctly?

**2. Determinants of (Non-)Utilization**

- What led you to (or kept you from) using [type of service]?
- What factors made it challenging—or easier—for you to consider, seek, or use [type of service]?
- What steps, if any, have you already taken toward initiating or arranging [type of service]?
- Who or what supported you—or might support you—in doing so?
- Who else, if anyone, was involved in the decision to use or not use [type of service]?

**3. Need-Congruent Mental Health Care Provision**

- Do you feel you had enough information and support to make an informed choice about [type of service]?
- If yes, what helped you? If no, what was missing? What would you have preferred to be different?
- Looking back, what kind of care or support would have best suited your needs?
- What alternative options to [type of service] do you know about?

**4. Stigmatization and Perceptions**

- What were your perceptions of [type of service] before you used or considered using it? Have these changed over time?
- When you think about seeking help for psychological difficulties, what thoughts, expectations, or concerns come to mind?
- How much time passed before you made the decision to seek out [type of service] (if applicable)?
- Have you talked to friends about seeking help for mental health concerns, or would you consider doing so? What might encourage or discourage you from discussing this with them?
