# Supplementary material for: No utilization without subjective need – a mixed-methods study on guideline-compliant mental health care for individuals with depressive symptoms
Source: BMC Health Serv Res. 2026 Sep 10;26:1261. doi: 10.1186/s12913-026-15562-3 (PMC13563709; doi:10.1186/s12913-026-15562-3)
Supplement: Supplementary file 2 [file 12913_2026_15562_MOESM2_ESM.docx]

## Supplementary file 2

***Table S1.*** *Schedule of enrollment and assessment providing references and adaptations.*

|  | | | | Study period |  |
| --- | --- | --- | --- | --- | --- |
| Timepoint | | | | T0 | T1 |
|  | English version^a^ | German version^a^ | Adaptations | Baseline  Telephone interview | 12 months  Telephone interview |
| Variables/Measures |  |  |  |  |  |
| Age |  |  |  | X |  |
| Sex |  |  |  | X |  |
| Migration background |  |  |  | X |  |
| Socioeconomic status | n.a. | [1]^b^ |  | X | Changes are queried |
| PHQ-9 (depression  symptomatology) | [2] | [3] | none | X ^c^ | X |
| DIA-X-12/M-CIDI  (diagnosis/objective need) | n.a. | [4, 5] | none |  |  |
| Subjective need for   Pharmacotherapy and/or   counselling/psychotherapy | [6] | Own translation^b^ | Answer options were adapted to allow for the evaluation of subjective needs regardless of current treatment status: “I would like to use this offer of help,” “I do not want to use this offer of help,” “I already use this offer of help and I have also wanted to use it,” and “I am already using this offer of help, but I did not want to use it.” | X | X |
| Risk of mental comorbidity  index (GAD-7, AUDIT-C,   PHQ-15, SSD-12) | *GAD-7:* [7]  *AUDIT-C:* [8, 9]  *PHQ-15:* [10] *SSD-12:* n.a. | *GAD-7:* [11] *AUDIT-C:* [12]  *PHQ-15:* [3]  *SSD-12:* [13, 14]^b^ | none | X | X |
| Mental health service use | n.a. | [15]^b^ | Response options were adapted on the basis of the pretest results (e.g., established psychotherapist instead of psychotherapist).  *Medication treatment (open-ended question)*  *Inpatient facilities:*  “Psychiatric, psychotherapeutic or psychosomatic clinic or ward”, “Psychosomatic rehabilitation”, “Psychiatric, psychotherapeutic or psychosomatic day clinic”, “Other inpatient facilities namely”  *Outpatient facilities:*  “Psychiatric, psychotherapeutic or psychosomatic outpatient clinic”, “(Socio-)psychiatric service”, “established Psychiatrist/Neurologist”, “established Psychotherapist”, “General practitioner (for mental problems)”, “Counseling centers for” (to be specified), “Other outpatient facilities namely” | X^d^ | X^e^ |

^a^ Sources for validated measurements are provided if an English version is available; for demographic measures, please see the translations below.
^b^ Available upon request in German version. ^c^ Serves as eligibility screen at T0. ^d^ Lifetime, 12-month, and point prevalence of mental health care utilisation were
assessed. ^e^ 12-month and point-prevalence were assessed with specific time spans.

| 1.1 **Your sex?** | | | 1.2 **Your age?** | | |
| --- | --- | --- | --- | --- | --- |
| 🞎_1_ female | | | _____ years | | |
| 🞎_2_ male  🞎_3_ divers | | |  |  |  |
| 1.3 **What is your nationality?** | | | | | |
| 🞎_1_ German | | 🞎_2_ German emigrant  (year of emigration)  Ι__Ι__Ι__Ι__Ι | | | 🞎_3_ other nationality, specifically: ________________________ |
| In which country were you born? | 🞎_1_ Germany  🞎_2_ Other country, specifically: __________________________________________ | | | | |
| In which country were your parents born? | mother:  🞎_1_ Germany  🞎_2_ Other country, specifically:  ________________________ | | | father:  🞎_1_ Germany  🞎_2_ Other country, specifically:  ____________________________ | |

## Literature

1.	Lampert T, Kroll LE, Müters S, Stolzenberg H: **Messung des sozioökonomischen Status in der Studie zur Gesundheit Erwachsener in Deutschland (DEGS1)**. *Bundesgesundheitsblatt-Gesundheitsforschung-Gesundheitsschutz* 2013, **56**(5-6):631-636.

2.	Kroenke K, Spitzer RL, Williams JB: **The PHQ‐9: validity of a brief depression severity measure**. *Journal of general internal medicine* 2001, **16**(9):606-613.

3.	Gräfe K, Zipfel S, Herzog W, Löwe B: **Screening for psychiatric disorders with the Patient Health Questionnaire (PHQ). Results from the German validation study**. *Diagnostica* 2004, **50**(4):171-181.

4.	Wittchen H-U: **Reliability and validity studies of the WHO-Composite International Diagnostic Interview (CIDI): a critical review**. *Journal of psychiatric research* 1994, **28**(1):57-84.

5.	Wittchen H-U, Pfister H: **DIA-X-Interviews: Manual für Screening-Verfahren und Interview; Interviewheft**. *Interviewheft* 1997.

6.	McNab C, Meadows G: **The General-practice Users' Perceived-need Inventory (GUPI'): a brief general practice tool to assist in bringing mental healthcare needs to professional attention**. *Primary Care Mental Health* 2005, **3**(2):93-101.

7.	Spitzer RL, Kroenke K, Williams JBW, Löwe B: **A Brief Measure for Assessing Generalized Anxiety Disorder: The GAD-7**. *Archives of Internal Medicine* 2006, **166**(10):1092-1097.

8.	Bush K, Kivlahan DR, McDonell MB, Fihn SD, Bradley KA: **The AUDIT alcohol consumption questions (AUDIT-C): an effective brief screening test for problem drinking. Ambulatory Care Quality Improvement Project (ACQUIP). Alcohol Use Disorders Identification Test**. *Arch Intern Med* 1998, **158**(16):1789-1795.

9.	Saunders JB, Aasland OG, Babor TF, De La Fuente JR, Grant M: **Development of the Alcohol Use Disorders Identification Test (AUDIT): WHO Collaborative Project on Early Detection of Persons with Harmful Alcohol Consumption-II**. *Addiction* 1993, **88**(6):791-804.

10.	Kroenke K, Spitzer RL, Williams JB: **The PHQ-15: validity of a new measure for evaluating the severity of somatic symptoms**. *Psychosom Med* 2002, **64**(2):258-266.

11.	Löwe B, Decker O, Müller S, Brähler E, Schellberg D, Herzog W, Herzberg PY: **Validation and standardization of the Generalized Anxiety Disorder Screener (GAD-7) in the general population**. *Med Care* 2008, **46**(3):266-274.

12.	Moehring A, Rumpf H-J, Hapke U, Bischof G, John U, Meyer C: **Diagnostic performance of the Alcohol Use Disorders Identification Test (AUDIT) in detecting DSM-5 alcohol use disorders in the General population**. *Drug and Alcohol Dependence* 2019, **204**:107530.

13.	Toussaint A, Löwe B, Brähler E, Jordan P: **The Somatic Symptom Disorder - B Criteria Scale (SSD-12): Factorial structure, validity and population-based norms**. *Journal of Psychosomatic Research* 2017, **97**:9-17.

14.	Toussaint A, Murray AM, Voigt K, Herzog A, Gierk B, Kroenke K, Rief W, Henningsen P, Löwe B: **Development and Validation of the Somatic Symptom Disorder–B Criteria Scale (SSD-12)**. *Psychosomatic Medicine* 2016, **78**(1):5-12.

15.	Jacobi F, Mack S, Gerschler A, Scholl L, Höfler M, Siegert J, Bürkner A, Preiss S, Spitzer K, Busch M *et al*: **The design and methods of the mental health module in the German Health Interview and Examination Survey for Adults (DEGS1‐MH)**. *International journal of methods in psychiatric research* 2013, **22**(2):83-99.
